# Toward Development of a Higher Flow Rate Hemocompatible Biomimetic Microfluidic Blood Oxygenator

**DOI:** 10.3390/mi12080888

**Published:** 2021-07-28

**Authors:** Jose Santos, Else M. Vedula, Weixuan Lai, Brett C. Isenberg, Diana J. Lewis, Dan Lang, David Sutherland, Teryn R. Roberts, George T. Harea, Christian Wells, Bryan Teece, Paramesh Karandikar, Joseph Urban, Thomas Risoleo, Alla Gimbel, Derek Solt, Sahar Leazer, Kevin K. Chung, Sivaprasad Sukavaneshvar, Andriy I. Batchinsky, Jeffrey T. Borenstein

**Affiliations:** 1 Draper, Cambridge, MA 02139, USA; jsantos@draper.com (J.S.); wlai@draper.com (W.L.); bisenberg@draper.com (B.C.I.); dlewis@draper.com (D.J.L.); dlang@draper.com (D.L.); dsutherland@draper.com (D.S.); cwells@draper.com (C.W.); bteece@draper.com (B.T.); pkarandikar@draper.com (P.K.); jurban@draper.com (J.U.); thomas.risoleo@gmail.com (T.R.); agimbel@draper.com (A.G.); 2 Autonomous Reanimation and Evacuation (AREVA) Research Program, The Geneva Foundation, Brooks City Base, San Antonio, TX 78006, USA; TRoberts@genevausa.org (T.R.R.); gharea@genevausa.org (G.T.H.); abatchinsky@genevausa.org (A.I.B.); 3 Thrombodyne, Inc., Salt Lake City, UT 84103, USA; derek@thrombodyne.com (D.S.); sp@thrombodyne.com (S.S.); 4 Department of Medicine, F. Edward Hebert School of Medicine, Uniformed Services University of the Health Sciences, Bethesda, MD 20814, USA; sahar.leazer.ctr@usuhs.edu (S.L.); kevin.chung@usuhs.edu (K.K.C.)

**Keywords:** microfluidics, oxygenator, hemocompatibility, extracorporeal, lung

## Abstract

The recent emergence of microfluidic extracorporeal lung support technologies presents an opportunity to achieve high gas transfer efficiency and improved hemocompatibility relative to the current standard of care in extracorporeal membrane oxygenation (ECMO). However, a critical challenge in the field is the ability to scale these devices to clinically relevant blood flow rates, in part because the typically very low blood flow in a single layer of a microfluidic oxygenator device requires stacking of a logistically challenging number of layers. We have developed biomimetic microfluidic oxygenators for the past decade and report here on the development of a high-flow (30 mL/min) single-layer prototype, scalable to larger structures via stacking and assembly with blood distribution manifolds. Microfluidic oxygenators were designed with biomimetic in-layer blood distribution manifolds and arrays of parallel transfer channels, and were fabricated using high precision machined durable metal master molds and microreplication with silicone films, resulting in large area gas transfer devices. Oxygen transfer was evaluated by flowing 100% O_2_ at 100 mL/min and blood at 0–30 mL/min while monitoring increases in O_2_ partial pressures in the blood. This design resulted in an oxygen saturation increase from 65% to 95% at 20 mL/min and operation up to 30 mL/min in multiple devices, the highest value yet recorded in a single layer microfluidic device. In addition to evaluation of the device for blood oxygenation, a 6-h in vitro hemocompatibility test was conducted on devices (*n* = 5) at a 25 mL/min blood flow rate with heparinized swine donor blood against control circuits (*n* = 3). Initial hemocompatibility results indicate that this technology has the potential to benefit future applications in extracorporeal lung support technologies for acute lung injury.

## 1. Introduction

The rapidly increasing prevalence of chronic lung diseases such as Chronic Obstructive Pulmonary Disease (COPD) [1] and unpredictable and potentially overwhelming outbreaks of acute infectious illnesses such as swine flu [2] and COVID-19 [3] highlight the urgent need for improved treatments for respiratory insufficiency and respiratory failure versus the current standard of care, mechanical ventilation [4]. Invasive mechanical ventilation (MV) is administered to several hundred thousand patients each year, supporting oxygenation and carbon dioxide removal in patients with cardiopulmonary failure and acute respiratory distress syndrome (ARDS) from a variety of causes. However, MV is associated with mechanical damage to already fragile lungs (barotrauma), risk of ventilator-associated pneumonia or other infections due to microbial entry during the procedure, and a complex array of mechanisms of inflammatory and biochemically-induced damage to the lungs, termed biotrauma [5]. These factors, along with the challenges associated with the use of MV to effectively treat emerging pandemic infections such as COVID-19 [6,7], have dramatically increased the level of interest in Extracorporeal Membrane Oxygenation (ECMO) [8], during which blood is circulated through an external circuit where gas exchange takes place in an artificial lung, allowing ventilator support to be reduced or, in rare cases, avoided.

Early and successful application of ECMO for neonates [9,10,11,12] has been followed by expanded use in adult populations, demonstrating a survival benefit in the 2009 swine flu epidemic [13] and suggesting a benefit in the widely-cited CESAR trial [14]. However, ECMO administration carries its own risks, largely related to the complexity of the blood circuit and the risk of clotting and bleeding [5,15]. Limitations in the efficiency of the gas transfer process, a requirement for fairly vigorous gas/blood mixing particularly in extracorporeal carbon dioxide removal (ECCO2R) [16], and complications involving anticoagulation, heparin dosing, and thrombus formation, have all contributed toward efforts to look for safer and more efficacious advances in ECMO technology.

The emergence of microfluidics technology presents an enormous opportunity to address many of the fundamental challenges in ECMO, due to the ability of microfluidics to fashion gas transfer membranes thinner than hollow fiber membrane oxygenators (HFMO), shallower blood channels, and potentially to better recapitulate the smooth and gentle manner in which blood flows through small vessels in the lung during the physiological gas transfer process [17,18,19]. Over the past decade, tremendous progress has been made by a number of groups in the development of microfluidic oxygenators, leveraging advances in computational designs, microfabrication techniques, and biomaterials technologies toward prototype devices that have been tested in vitro and in vivo in a large number of proof of concept studies [17,18,19,20,21,22,23,24,25,26,27,28,29,30].

Early results from prototyped small-scale microfluidic oxygenator blood gas transfer in the laboratory and in initial animal experiments [31,32] have been promising, but progress in advancing microfluidic prototypes toward a scale large enough for large animal studies and human clinical trials has been limited. In most microfluidic designs blood flow patterns remain highly non-physiological due to limitations in the ability of photolithography-based fabrication techniques to reproduce critical aspects of the architecture of the microcirculation, including smooth, rounded channels and gradually tapered transition regions at branch points between vessels. Further, the ability of microfluidics to scale these systems toward clinical blood flow rates is unclear [33,34], and therefore no devices with blood flow rates higher than 10 mL/min for a single microfluidic layer have been achieved.

In light of these considerations, we report on two principal innovations in the development of microfluidic oxygenators. The first is the use of fully three-dimensional microfluidic architectures to create the blood flow network: the microfabrication approach here enables smoothly varying depths as well as widths through the use of precision computer numerical control (CNC) machining to create master molds for microreplication. This approach enables better mimicry of physiological branching structures than conventional lithographic techniques that limit feature variations to the width and length axes in a planar network. The second significant advance reported here is a larger scale design that supports blood flow rates up to 30 mL/min in a single microfluidic layer, with blood gas transfer testing in porcine blood demonstrating high oxygen transfer rates. Prior efforts in microfluidic oxygenators have been limited to flow rates as high as only 10 mL/min [31] within a single layer, rendering it difficult to scale devices to clinically relevant blood flows without requiring an inordinate number of layers in a stacked device. This increase to 30 mL/min represents an important step toward clinically relevant flow rates per layer that can lead to practical oxygenator designs for clinical implementation.

The abovementioned advances in scaling, along with the proposed improvements in hemocompatibility, represent promising steps toward the ultimate clinical utilization of the microfluidic oxygenator technology reported here. Initial hemocompatibility studies of these devices in heparinized blood show stable performance in an in vitro circuit for up to 6 h, far longer than has been reported previously. These initial results suggest a developmental path for microfluidic oxygenators toward higher flow, hemocompatible operation in a variety of clinical settings. Early application of the technology may be realized in lower flow settings such as neonatal or pediatric use, or for adult respiratory support either as a carbon dioxide removal treatment or in conjunction with lower flow conventional respiratory support to minimize the deleterious effects associated with high oxygen/pressure ventilation. Ultimately, the objective of this research is to establish a safer and simpler technology for ECMO, enabling improved patient outcomes and broader access to the treatment to address the urgent need for new approaches for respiratory support.

## 2. Design and Experimental Methods

### 2.1. Device Design

The microfluidic oxygenator device is comprised of three layers as described earlier [19,23], one containing a connected network of oxygen gas-carrying channels, across from another layer consisting of a network of branching blood-carrying channels, separated by a thin non-porous gas-permeable Poly (DiMethylSiloxane) (PDMS) membrane. The vascular network layer has been carefully designed to minimize shear stress on the blood by creating a flow path that maintains laminar flow with relatively constant shear and minimal disruptions to the blood streamlines as it is carried from the inlet through a distribution manifold and into a parallel array of gas transfer channels [32].

The basic design of this device has been described previously [32]; here, the Draper team has increased the depth of the blood channels and modified the blood distribution manifold to accommodate the deeper channels, for the purpose of significantly reducing the blood pressure drop across the network while maintaining efficient oxygen transfer across the membrane. Briefly, the device design comprises 176 parallel blood channels, each of width 500 microns and separated by a 500-micron wall, a channel depth of 200 microns, and an overall channel length of 15.1 cm. A similar approach is invoked for the oxygen network design, where the oxygen network is oriented perpendicular to the vascular channel array. Channel depth is a critical parameter that drives many of the key performance metrics of the oxygenator. Shallower channels provide more efficient oxygen transfer, since the distance oxygen must travel from the source through the blood represents a significant limitation in transfer efficiency. However, shallower channels also increase the blood pressure drop across the device, raising the resistance the blood pump encounters for a given blood flow rate. We selected 200 microns for the channel depth to balance the tradeoff between oxygen transfer efficiency and blood pressure drop.

The distribution manifold carrying blood into and out of the parallel array of blood channels is designed to distribute blood evenly across each of the channels while avoiding sharp corners and sudden transitions at junctions and branch points and where the width or depth of the channels changes. As described in the Section 2.2 that follows, the master molding technology utilizes precision machining techniques that enable the depth and width of features to vary smoothly from one diameter to another, unlike conventional photolithographic means for producing masters for microfluidic molding. This design enables the blood distribution manifolds to accommodate the higher flows necessary to carry blood to and from multiple transfer channels to be deeper than the corresponding transfer channel, thereby avoiding the need for excessively wide trunk regions in the network. A guiding principle for the design of these distribution networks is Murray’s Law [35], which is based upon observations regarding the morphometric features of physiologic circulation networks. Murray’s law states that at a bifurcation, the third power of the parent channel diameter is equal to the sum of the third power values of the two daughter channels. This law has been adapted for rectangular channels in microfluidic networks [36] and provides a basis for physiologically relevant and biomimetic designs. Further, the constant shear behavior of flow networks based on this approach follows naturally from the Murray’s Law design principles.

### 2.2. Layer Fabrication

**Figure 1:**
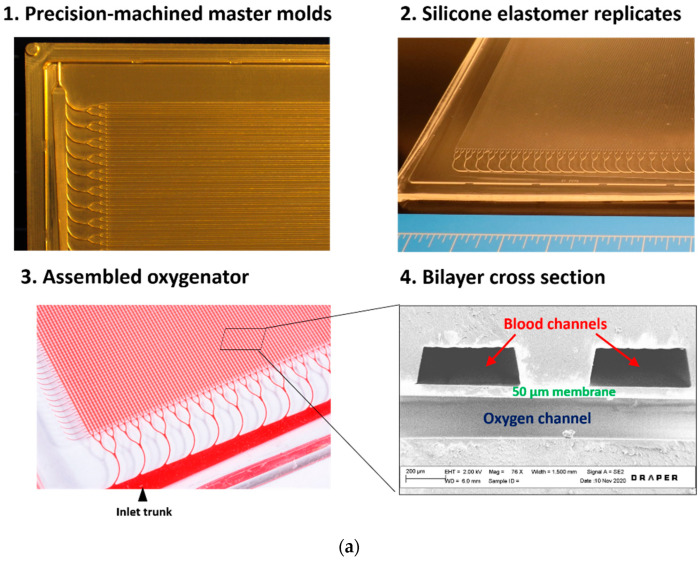

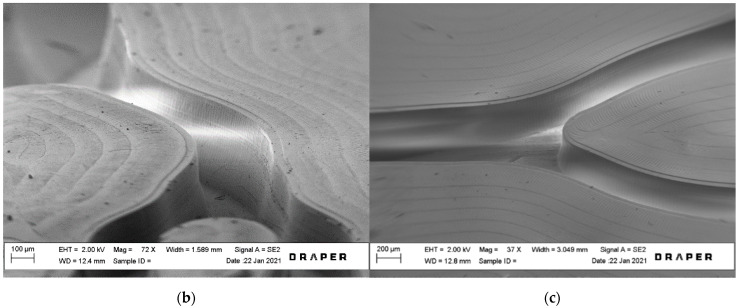
(**a**) Images of fabrication components. (1): Precision machined aluminum master mold section showing blood distribution manifold branching into parallel channel array; (2): Silicone elastomer replica constructed from NuSil; (3): Assembled microfluidic vascular and oxygen layers bonded to cast membrane; (4): SEM image of a cross-section showing blood channels and an oxygen channel separated by a 50-micron gas transfer membrane. (**b**) SEM images of branching channel junction with varying channel depth. (**c**) SEM of smooth branch point in blood channel layer.

Aluminum master molds were precision-machined using a computer numerical control (CNC) milling machine at Draper, programmed according to the layer designs described above. These served as templates for the device layer fabrication of the vascular and oxygen layers. Specific geometries were invoked for oxygen and vascular layers of the microfluidic oxygenator, as seen in Figure 1, and as described elsewhere [32]. The vascular layer comprised an array of 176 parallel 500 µm wide and 200 µm deep channels, 15.1 cm in length. The oxygen layer also comprised an orthogonal array of 500 µm wide and 200 µm deep channels.

**Figure 2:**
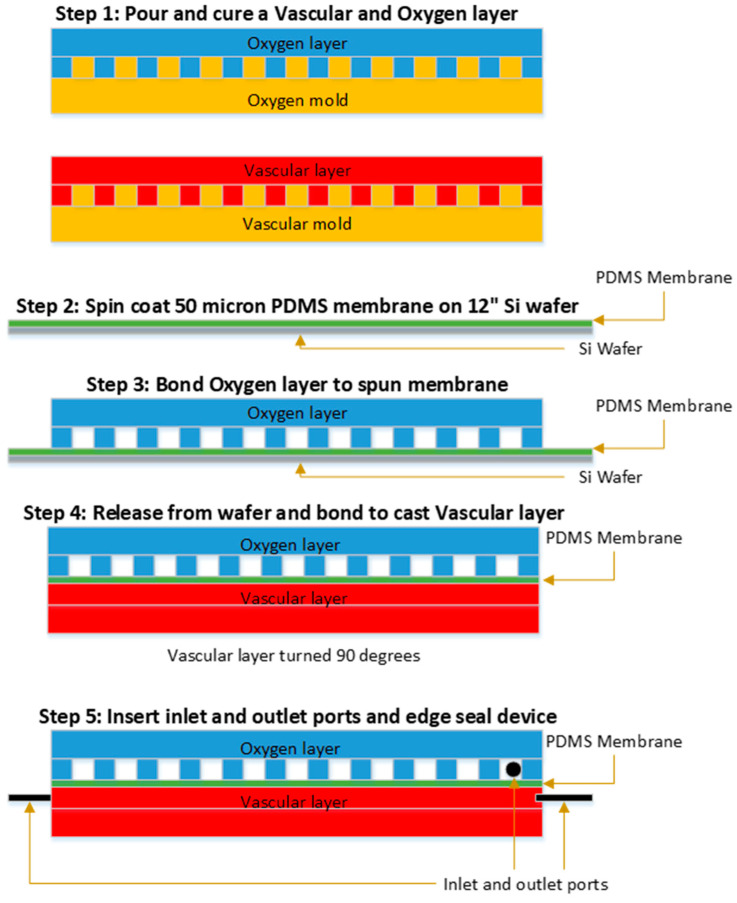
Fabrication process for microfluidic oxygenator. Master molds for vascular and oxygen are shown in gold, while oxygen layers are shown in blue and vascular layers in red. The NuSil membrane is shown in green. Step 1: Cast NuSil vascular and oxygen layers against master molds, creating open channels in the NuSil layers via microreplication from the gold-coated micromachined molds. Step 2: Spin cast Nusil membrane against a polished silicon wafer. Step 3: Bond oxygen layer to spin-cast membrane. Step 4: Bond vascular layer to other side of membrane, creating a single layer sandwich structure for gas transfer. Step 5: Insert tubing connections (shown at left and right in the oxygen layer, in and out of plane in the vascular layer) and apply edge seal to the assembled sandwich structure.

The fabrication process is illustrated in Figure 2. Following precision machining, Al molds were sputtered (KDF Sputterer, Rockleigh, NJ, USA) with a 500 Å layer of Au to promote the release of the casted PDMS layers from the mold surface. Vascular and oxygen layers of the device were made from NuSil (MED-6015, NuSil, Carpinteria, CA, USA), a two-part transparent silicone elastomer (PDMS) comprising an elastomer and a curing agent. The silicone elastomer and curing agent were mixed at a 10:1 ratio by weight using the SpeedMixer DAC 600.1 FVZ (FlackTek, Landrum, SC, USA). The NuSil was mixed at 1000 RPM for 15 s, followed by mixing at 2000 RPM for 1 min. A mass of 300 g of the mixed NuSil was poured onto the Al mold for the vascular layer, and 110 g was poured into the oxygen layer mold; poured molds were placed in a desiccator to degas for 45 min. Degassed layers were cured at 65 °C for a minimum of 3 h. Cured vascular and oxygen layers were removed from the molds and cut to size, with tabs to support the insertion of tubing in the final device once assembled. The final layer footprint was approximately 12 cm × 16 cm.

### 2.3. Membrane Fabrication

Prior to being sandwiched between vascular and oxygen channel layers, a thin, non-porous membrane was fabricated by spin-coating a polished 300 mm diameter silicon wafer using a Cee 300X precision spin coater (Brewer Science, Rolla, MO, USA) at 1100 rpm for 60 s with a 500 rpm/s ramp up rate. The target membrane thickness was 50 μm. The silicon wafer was coated with Trichlorosilane (Aldrich, St. Louis, MO, USA) to allow the spun NuSil film to be easily released from the surface of the wafer. Once coated, the membranes were allowed to settle at room temperature for 10 min, then placed in an oven at 65 °C for at least 1.5 h.

### 2.4. Device Assembly

Device layers were bonded together by applying a thin layer of adhesive using methods previously described [32]. Briefly, a thin layer of DOWSIL 3140 RTV (Dow Silicone Corporation, Midland, MI, USA) adhesive was rolled out on a flat surface and was transferred to the oxygen layer using an ink roller. The layer was then placed on the spin-coated membrane while it remained on the wafer, and the bond was cured overnight at 65 °C. This bonding method was repeated to attach the vascular layer to the other side of the membrane. Prior to this second bond, the oxygen layer and membrane were removed from the Si wafer, first by cutting around the layer and then gently peeling the casted layer and membrane away from the wafer surface. The vascular layer and oxygen layer were aligned with one other to create a perpendicular arrangement of the oxygen relative to the blood channels. The assembled stack was placed in an oven set to a temperature of 65 °C for at least 1.5 h.

Tubing and interconnections to the layer channels were assembled with 1/32″ ID Tygon tubing (Cole Palmer, Vernon Hills, IL, USA), 1/32″ ID stainless steel tubing for support (New England Small Tube, Litchfield, NH, USA), and 1/16″ ID barb hose to Luer lock connections (Cole Palmer, Vernon Hills, IL, USA). The Luer lock connections were interfaced with blunt-tipped syringe needles during testing. The assembled tubing was then inserted into the inlets and outlets of the assembled oxygen, vascular, and membrane stack. The edges and tubing interfaces of the assembled stack were coated with a thin layer of uncured NuSil before being placed in an oven at 65 °C for at least 3 h to cure. The assembled and cured device was briefly tested for leaks before all oxygen transfer or blood health testing.

### 2.5. Oxygen Transfer Testing

**Figure 3:**
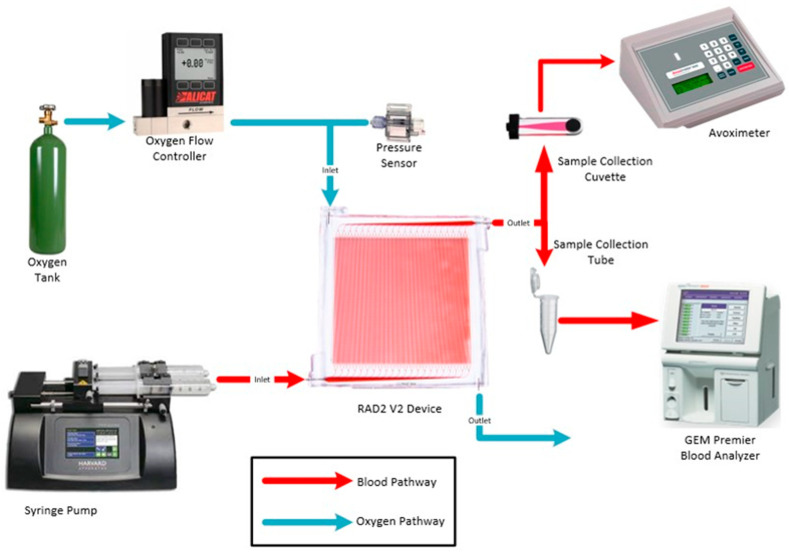
Schematic illustrating test circuit for blood gas transfer testing, showing various components in the test apparatus. The oxygen source bottle is on the left (pure oxygen), with the O_2_ flow metered by the oxygen flow controller, and pressure measured by the sensor as shown. The RAD2v2 device is in the center, with the syringe pump delivering blood as shown on the left. Blood exits the device on the right and is measured for blood gas parameters with the hemoximeter (upper right) and blood gas analyzer (lower right), providing data including hematocrit, dissolved oxygen concentration, and hemoglobin-bound oxygen.

The experimental setup is illustrated in Figure 3. The setup consists of a Harvard Apparatus syringe pump (Harvard Apparatus, Holliston, MA, USA), a pressure sensor (Pendotech, Princeton, NJ, USA), two pressure controllers (Alicat, Tucson, AZ, USA), heated blood rocker (ThermoScientific, Waltham, MA, USA), and the assembled device. The syringe pump controlled the blood flow into the device. The pressure sensor was used to monitor the inlet pressure in the vascular channel and the pressure drop in mmHg across the device and the tubing connections. The two pressure controllers controlled the inlet and outlet pressure of the oxygen channel. The vascular channel layer of the assembled device was purged and primed with ethanol to remove air bubbles from the device. Connections between the syringe, the device, pressure sensors, and pressure controllers were all Luer lock connections. Connections to the vascular layer were made using the wet-to-wet connection technique with an EtOH prime followed by a saline prime to ensure no air bubbles were introduced in the line.

A pressure controller was connected in-line between the oxygen tank and the oxygen inlet of the device. This controller controlled the oxygen flow out of the tank into the device inlet. The blood was tested after it had been passed through the device using a Blood Gas Analyzer (ABG) (Instrumentation Laboratory, Bedford, MA, USA), and the Avoximeter (ITC, Edison, NJ, USA). The ABG was used to measure the dissolved oxygen content in the blood before and during the experiment. The Avoximeter measures the oxyhemoglobin content (percent of hemoglobin bound to oxygen) before and during the experiment. Experiments were performed using porcine blood collected with the administration of the anticoagulant citrate-phosphate-dextrose (CPD) (Lampire Biological Laboratories, Pipersville, PA, USA). The blood was heated up to 37 °C and conditioned to venous conditions for pH, O_2_, and CO_2_ concentrations (pH = 7.31, pO_2_ = 30 mmHg, pCO_2_ = 41 mmHg). The blood was placed in a heated rocker to prevent clotting, and to hold the operating temperature steady at the target level.

Blood was drawn into a syringe at the start of each experiment. The blood in the syringe was tested with one ABG reading and three Avoximeter readings before flowing through the device. This established a baseline for the blood going into the device. The oxygen pressure controller was set to provide a 100 mL/min oxygen flow rate. Blood flow rates included 10, 20, and 30 mL/min through a single layer of the microfluidic oxygenators. The flow rates were held for durations ranging from 2 to 4 min. Flow rates were not randomized, but fresh venous blood was introduced prior to each measurement. Samples from the blood outlet were taken for measurements using the ABG and the Avoximeter. One ABG reading and three Avoximeter readings were taken for each sample collected at the outlet. These measurements were used to calculate the transfer of oxygen from the oxygen compartment into the blood channel compartment. The volume percent oxygen transfer calculation is covered in detail elsewhere [23]. We correct the volume percent transfer to account for variations in hematocrit (standardized to 12 g/dL) and in the ambient pressure and temperature, as described elsewhere [18].

### 2.6. Hemocompatibility Studies

The AREVA lab carried out hemocompatibility testing of the RAD2v2 oxygenator in a 6-h ex vivo blood circulation model using heparinized swine blood. The blood (1 L) was collected from swine donors at the end of an unrelated study, according to an approved animal use protocol. Use of blood was in accordance with animal use principles of “reduction” and “refinement”, and also enabled assessment of hemocompatibility using “injured” blood, such as would be the case during clinical utilization of extracorporeal organ support devices. Blood was collected into unfractionated heparin (Heparin Sodium Injection, Sagent Pharmaceuticals; Schaumburg, IL, USA) to a target activated clotting time (ACT) of 200–300 s, with additional heparin boluses added (100 U per bolus) throughout circulation to maintain the ACT in the target range. The collected volume of 1 L of blood was divided evenly to enable the testing of two circuits simultaneously (500 mL/circuit) and was maintained at 37 °C.

**Figure 4:**
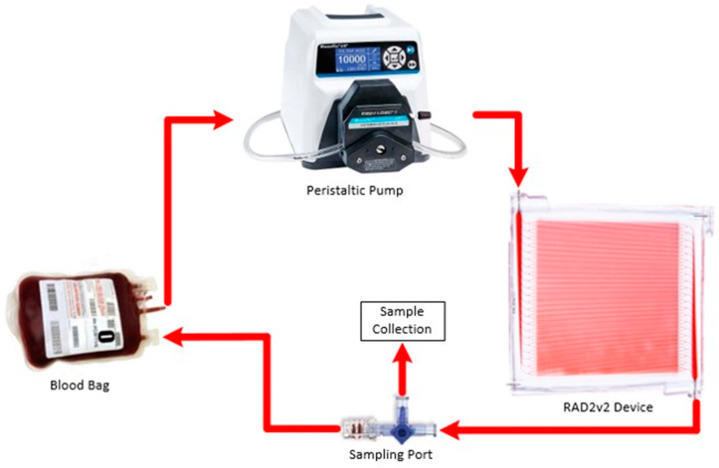
Schematic of experimental circuitry and monitoring setup. Blood is stored in the blood bag and flows through the Pump, RAD2v2 oxygenator then back to the blood bag. Flowmeters are clamped onto the tubing pre and post RAD2v2 oxygenator (F1 and F2). Pressure monitoring through a Luer port pre and post RAD2v2 oxygenator (P1 and P2).

A circuit consisted of a blood reservoir, RAD2v2 oxygenator, 1/8″ inner diameter (ID) connective tubing, and a peristaltic pump (Masterflex Pump System, Cole Parmer; Vernon Hills, IL, USA) (Figure 4). Connective tubing leading into and out of the RAD2v2 oxygenators was 1/16″ ID. Additionally, control circuits (*n* = 3) consisting of blood reservoir, tubing connections, peristaltic pump, but no in-line oxygenators were assessed to differentiate the effects of the circuit components relative to the RAD2v2 oxygenator specifically. Two RAD2v2 oxygenators were tested simultaneously in separate circulation loops, or one oxygenator was tested alongside a control circuit. Circuits were primed with normal saline prior to the start of the blood circulation. Blood flow rate, standardized to 25 mL/min, was monitored using Transonic Systems (Ithaca, NY, USA) PXL Clamp-On flow sensors, and pressure was monitored using a TruWave pressure transducer (Edwards Lifesciences; Irvine, CA, USA). The number of pump revolutions per minute to maintain flow, as well as the heparin requirements to maintain target ACT, were recorded. Blood flow was set to 25 mL/min for hemocompatibility testing in order to evaluate blood at a flow rate relevant for oxygen transfer; as noted in the Results section, the RAD2v2 is projected to achieve roughly 3.3 volume percent oxygen transfer at this flow rate. Hourly blood samples were collected for blood gas assessment (GEM Premier 5000, Instrumentation Laboratories; Bedford, MA, USA) to ensure that blood pH, partial pressure of oxygen, and partial pressure of carbon dioxide all remained within physiological limits. At the start of circulation (baseline), and at 3 h and 6 h circulation time, complete blood count (CBC) was measured via hematology analyzer (ADVIA 2120, Siemens; Munich, Germany) and concentration of plasma free hemoglobin (fHb) was assessed via spectrophotometric methods [37]. Additionally, blood was collected for coagulation assessment using thromboelastography (TEG) with citrated kaolin test (TEG 5000, Haemonetics Corp.; Boston, MA, USA), platelet aggregometry (Chrono-Log Corp.; Havertown, PA, USA) with collagen (3.2 µg/mL; Helena Laboratories; Beaumont, TX, USA) for activation, and flow cytometry (Cytoflex, Beckman Coulter Life Sciences; Indianapolis, IN, USA) to evaluate platelet surface expression of P-selectin (CD62P, activated platelet marker) and phosphatidyl serine (PS, procoagulant surface marker). For flow cytometry analysis, as previously reported, 5 µL of EDTA anticoagulated whole blood was incubated for 20 min with monoclonal antibodies for CD61 (platelet glycoprotein IIIa) (Bio-Rad; Hercules, CA, USA), CD62-RPE (Bio-Rad; Hercules, CA, USA) and bovine lactadherin-FITC (Haematologic Technologies, Inc.; Essex Junction, VT, USA) at room temperature. Samples were then diluted using Hank’s buffered salt solution and analyzed immediately with gating of the platelet population (CD61 positive population) [38]. Results are expressed as a percentage of P-selectin positive and PS positive platelets. Statistical analysis was performed using SAS 9.4 (Cary, NC, USA). All tests were two-sided with alpha <0.05 for significance. A Shapiro–Wilk test was first conducted to assess the distribution of the data for normality. If skewed, the data were then transformed, or a nonparametric version of the test was used. Groups were independently tested using one-way mixed models with repeated measures and Dunnett adjustment, to evaluate changes over time from the start of circulation. Between-group differences were examined using a two-way mixed model with repeated measures and Tukey adjustment.

## 3. Results and Discussion

### 3.1. Blood Gas Oxygen Transfer

**Figure 5:**
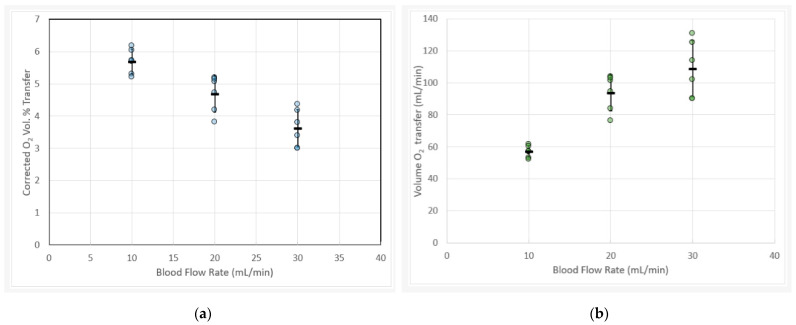
(**a**) Oxygen transfer rates versus blood flow rates for six RAD2v2 devices showing oxygen transfer (volume percent, defined as mL O_2_/min divided by blood flow rate (mL/min) corrected for hemoglobin and STP.) At each blood flow rate, each device was tested three times and the average recorded. The standard deviation reflects the variation between the six devices tested. Note that the volume percent at 30 mL/min blood flow corresponds to an increase in oxygen saturation from 75 to 95%. (**b**) Data from Figure 5a plotted as volume rate of oxygen transfer versus blood flow rate.

Oxygen transfer was evaluated in an in vitro test setup at Draper, using CPD-treated porcine whole blood as described in the Methods section, utilizing measurement techniques that directly obtain readings of hematocrit, dissolved oxygen concentration, and hemoglobin-bound oxygen concentration. A total of six RAD2v2 devices were tested across blood flow rates ranging from 10–30 mL/min in the circuit shown in Figure 3. Each device was tested three times at each blood flow rate, with volume percent oxygen transfer calculated as described elsewhere [23], where it is noted that 5 volume percent transfer corresponds to raising venous blood from 65% oxygen saturation to 95%, while a 3.3 volume percent transfer raises oxygen saturation from 75% to 95%. Corrected volume percent oxygen transfer is plotted against blood flow rate in Figure 5a, where the average (*n* = 6, two per test day and each test day with a different lot of porcine blood) is shown along with error bars indicating one standard deviation above or below the mean. The procedure used to correct the volume percent oxygen transfer was described in the Methods section. In Figure 5b, the volume rate of oxygen transfer versus blood flow is plotted in a similar manner. Note that oxygen transfer at 30 mL/min blood flow rate exceeds 3.3 volume percent, the highest level yet reported for a single layer microfluidic oxygenator.

**Figure 6:**
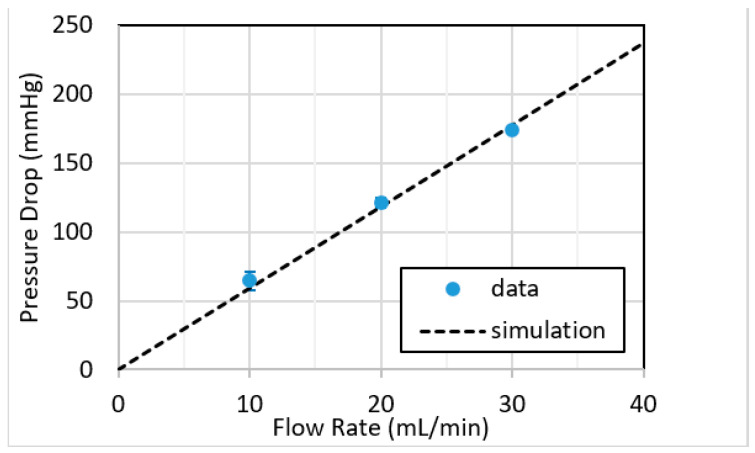
Measured pressure drop across RAD2v2 devices (*n* = 4) in mmHg as a function of blood flow rate in mL/min. Note that at 25 mL/min blood flow rate, the measured pressure drop is approximately 150 mm Hg, a value that includes the pressure drop of the device itself as well as the inlet and outlet tubing lengths connecting the oxygenator prototype to the circuit. Error bars indicate one standard deviation around the mean.

The pressure drop across the blood circuit was also monitored as described in the Methods section. The pressure drop included the device layer and the tubing connections. The pressure was recorded continuously during blood flow, and averages at each flow rate in four devices (shown in Figure 6) were compared with calculated values as described previously [23]. The experimental level of the pressure drop included the drop in the tubing, which was calculated at 25 mL/min to be 57 mm Hg. Note that the pressure drop measured at 25 mL/min blood flow, shown in Figure 6**,** averages approximately 150 mm Hg, and therefore if the contribution from the tubing lengths at the inlet and outlet of the device (57 mm Hg) is accounted for, then the actual device pressure drop is approximately 93 mm Hg. This level, while far lower than previous microfluidic oxygenators we have reported [23], is still somewhat elevated relative to optimal pressure drop in an ECMO circuit, and therefore future designs will aim to address this aspect.

### 3.2. Hemocompatibility Evaluation

**Figure 7:**
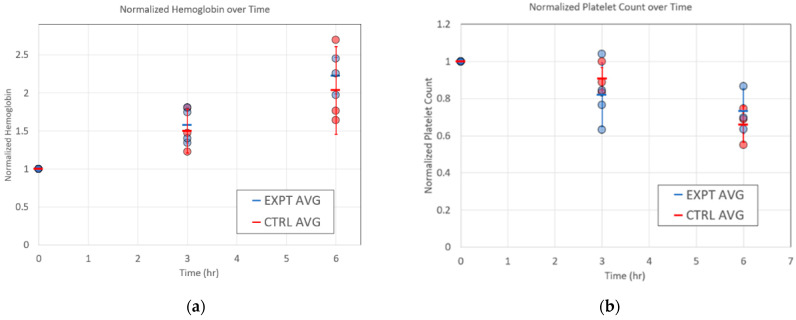
(**a**) Measurement of normalized plasma-free hemoglobin for the RAD2v2 microfluidic oxygenators (blue, *n* = 5) and control circuits (red, *n* = 3), showing standard deviations. (**b**) Normalized platelet count for microfluidic oxygenators (blue) versus control circuits (red). Three microfluidic devices completed the 6 h study, while two devices were terminated after 1.5 h and 3.5 h due to circuit occlusion.

AREVA labs conducted 6 h ex vivo hemocompatibility testing of the RAD2v2 devices relative to control circuits (no membrane in line) at 25 mL/min standardized blood flow rate. In these tests, three of the five RAD2v2 devices tested remained patent for 6 h circulation time, while two devices became occluded causing early study termination at 1.5 h and 3.5 h. All control circuits remained patent. Total heparin administered to maintain the target ACT of 200–300 s was not significantly different between groups (Control = 320 ± 150 U, RAD2v2 = 520 ± 20 U; *p* = 0.567). A summary of hemocompatibility and coagulation results is shown in Table 1. Of note, similar peristaltic pump revolutions per minute were required in both groups to maintain the blood flow rate at 25 mL/min. White and red blood cell counts were similar between groups and were not significantly reduced from the baseline/start of circulation levels. Interestingly, via thromboelastography, the time to the start of clot formation, or TEG Reaction Time (R), was significantly reduced in the RAD2v2 group at the start of blood circulation; and reduced further in both groups throughout the duration of circulation time. The initial clot formation rate, or TEG Alpha, was significantly elevated in the RAD2v2 group compared to controls at all time points compared. Platelet aggregation with collagen stimulation gradually decreased throughout circulation time; however, when aggregation was normalized to the platelet count there was no difference. This suggests the reduced aggregation is due to a decrease in platelet count. The percent of P-selectin and PS positive platelets was numerically higher in the RAD2v2 group versus the control. Data for normalized pfHb and platelet count across the 6 h experiment are shown in Figure 7a,b, respectively. In both figures, normalization was performed by dividing the timepoint value by the baseline (i.e., *t* = 0) value, and standard deviations for each condition appear for each of the two parameters. Note that there are no statistically significant differences between microfluidic oxygenators and the control circuit for each of these two key parameters as measured by *t*-test assuming unequal variance. This is a positive sign regarding hemocompatibility of the devices in future in vivo testing, as pfHb levels are known to be an independent predictor of mortality during ECMO therapy [39]. Further, thrombus formation can be inferred from monitoring of platelet counts in the circuit [40], as levels of circulating platelets will drop as thrombi form in various regions of the circuit including the connectors and tubing and the microchannels of the microfluidic oxygenator. We monitored both pfHb and platelet counts in RAD2v2 devices as described below, as initial indicators of hemocompatibility in an in vitro circuit.

## 4. Discussion

Respiratory assist devices are commanding increasing attention as a means to treat severe hypoxia in patients suffering from ARDS associated with traumatic injuries as well as a number of pulmonary infections, most notably swine flu and COVID-19. Application of ECMO in these patient populations continues to advance as the technology has overcome many challenges associated with clotting and plasma leakage [41]. However, significant barriers to expanded use of ECMO technology remain, particularly associated with the complexity of the blood circuit and anticoagulant administration. In response to these challenges, microfluidic oxygenators have emerged as a compelling means to address many of the safety and efficacy-related concerns with current ECMO procedures, driven in particular by opportunities to better mimic blood flow patterns in the natural lung and thereby avoid high rates of thrombus formation and heavy reliance on anticoagulant administration [17,23,31].

A very powerful potential advantage of microfluidic oxygenators over HFMO is represented by the ability of a microfluidic blood flow design to entrain the blood within a closed channel network throughout its complete excursion through the cartridge. In HFMO, blood flows over oxygen-carrying hollow fiber mats or bundles, and the blood flow patterns are governed by the shape of the overall cartridge housing and the positioning of the fibers within it. This results in the presence of regions of varying flow and shear rates—some excessively high while others are undesirably low [42]. These variations in flow and shear are associated with coagulation-related events [43] and likely increase the need for anticoagulant administration. In microfluidic oxygenators, blood flow is completely constrained within a precisely defined closed microchannel network. As such, with a suitably defined network design, flow and shear rates within it can be made uniform throughout [44]. This feature enables the reduction of coagulation-related events in the oxygenator and compelled us to explore the gas transfer performance and hemocompatibility of a microfluidic oxygenator design inspired by these principles. However, these approaches have been gated by limitations in the ability of conventional microfluidic fabrication technologies to recapitulate key aspects of the microcirculation and by challenges in scaling the technology toward clinical blood flow rates.

Scaling of the microfluidic oxygenator technology is often considered in terms of two directions. A given blood-membrane-gas sandwich structure can be scaled vertically by stacking these sandwich structures and interconnecting them with manifolds to integrate the blood and oxygen flows, as has been described previously [23,25,31]. A principal challenge this approach faces is the need for a very large number of layers in the stack [33], resulting in complexity in the assembly process as well as large increases in the blood prime volume in manifold regions not associated with blood–gas transfer. Another approach is to increase the capacity in terms of blood flow rate per layer, which becomes challenging given the need to accommodate large flows in a single network of channels. These large flows can result in high blood pressure drops, exacerbating shear-induced damage to the blood, and can be challenging from a fabrication standpoint.

Here, we demonstrate the highest blood flow rates yet reported in a single layer microfluidic oxygenator, with a 30 mL/min maximum blood flow rate enabled by a parallel array of 200-micron deep, 15 cm long channels connected via a biomimetic blood distribution manifold. The physiological relevance of the microchannel network here is represented best by the branching architecture of the flow paths, which is governed by similar principles as in vivo vasculature. The channel depth and width are commensurate with intermediate vessel dimensions in the circulation. Multiple devices have been evaluated, demonstrating the reproducibility of the design from a gas transfer performance standpoint. The blood pressure drop at 25 mL/min blood flow rate has been evaluated at just under 100 mm Hg, a relatively low pressure drop for such a high flow rate per layer.

Hemocompatibility assessment of these microfluidic oxygenators extended out to 6 h of blood flow, far longer than in most previous evaluations of blood stability in an in vitro setting. Ideally, microfluidic oxygenators would be compared directly with HFMO devices; however, the microfluidic counterparts have not yet reached a scale where a direct comparison can be made. Here, we have compared several key blood health parameters between a set of RAD2v2 microfluidic oxygenators and control circuits and have demonstrated the promising performance of the microfluidic devices from a blood health standpoint. Hemolysis in the microfluidic devices is low, consistent with the biomimetic design and careful management of shear in the system. Platelet counts and clot strength remain relatively similar between the microfluidic oxygenators and control circuits; however, differences in heparin dosing render direct comparisons difficult. Hemocompatibility testing objectives were exploratory, as the study was not powered to assess the specific outcomes measures reported and should be interpreted in this context.

Taken together, the results reported here, as obtained in two independent labs at two separate locations, provide an extensive early assessment of potential functional and coagulation-related outcomes during microcirculation at the flow rates tested. This is of significant value toward informing the development of scaled devices and novel materials for next-generation extracorporeal organ support technology.

## 5. Conclusions

In this study, we describe the design, fabrication, and initial in vitro prototype testing of a microfluidic oxygenator configuration that supports 30 mL/min blood flow, a new high blood flow rate yet reported in a single layer microfluidic device. At Draper, multiple devices were evaluated for oxygen transfer, demonstrating reproducible performance across several devices, test days, and batches of porcine blood. Hemocompatibility studies run for up to 6 h in an in vitro circuit demonstrate that key parameters such as plasma-free hemoglobin and platelet concentration remain roughly equivalent to control circuits. This is a promising sign for the stability of these early prototypes—after additional research and development, in vivo performance will be assessed in large animal studies at the AREVA labs. This investigation provides a potential pathway toward scaling of microfluidic oxygenators toward clinical applications in neonatal and ultimately in adult critical care.

## Figures and Tables

**Table 1:** Hemocompatibility testing at AREVA in the control group versus identical setup with RAD2v2 Oxygenators in line (RAD2v2, *n* = 5).

Variable	Group	Baseline	3H	6H
Blood Flow Rate (mL/min)	RAD2v2	25 ± 1	25 ± 0	25 ± 0
	Control	26 ± 1	25 ± 1	25 ± 1
Blood Pump (revolutions/min)	RAD2v2	24 ± 1	25 ± 1	25 ± 1
	Control	24 ± 0	23 ± 1	23 ± 1
WBC Count (×10^3^/µL)	RAD2v2	11 ± 3	9 ± 3	6 ± 2
	Control	10 ± 1	10 ± 2	8 ± 2
RBC Count (×10^6^/µL)	RAD2v2	4 ± 1	4 ± 1	4 ± 1
	Control	4 ± 1	4 ± 1	4 ± 1
Hgb (g/dL)	RAD2v2	7 ± 2	7 ± 1	7 ± 1
	Control	8 ± 2	7 ± 1	7 ± 1
TEG Reaction Time (R, min)	RAD2v2	3.3 ± 0.2 *^,†^	2.7 ± 0.1 *	2.3 ± 0.2 *
	Control	4.0 ± 0.2 *	3.4 ± 0.5 *	3.0 ± 0.4 *
TEG Initial Clot Formation Rate (Alpha, deg)	RAD2v2	0.8 ± 0.0 ^†^	0.8 ± 0.0 ^†^	0.8 ± 0.0 ^†^
	Control	1.1 ± 0.1	1.3 ± 0.0	1.5 ± 0.4
TEG Clot Strength (MA, mm)	RAD2v2	82 ± 6	81 ± 6	78 ± 7
	Control	71 ± 8	72 ± 12	68 ± 13
Collagen-induced platelet aggregation (AUC)	RAD2v2	67 ± 36	43 ± 30 *	15 ± 18 *
	Control	58 ± 24	43 ± 18	21 ± 11
%P-Selectin Positive Platelets	RAD2v2	3 ± 1	5 ± 2	9 ± 4
	Control	2 ± 1	3 ± 1	2 ± 1
% PS Positive Platelets	RAD2v2	4 ± 2	6 ± 3	9 ± 5
	Control	2 ± 1	3 ± 1	5 ± 1

Results are reported as mean ± standard deviation. *: Indicates significant change from Baseline value; ^†^: Represents significant between-group difference. All tests were two-sided with *p* < 0.05 for significance. WBC, white blood cell; RBC, red blood cell; Hgb, hemoglobin; AUC, area under curve.

## Data Availability

The data presented in this study are available on request from the corresponding author. The data are not publicly available yet, as it is still being formatted in a manner that will render it easily accessible to general users.
